# Finite element analysis of a bone healing model: 1-year follow-up after internal fixation surgery for femoral fracture

**Published:** 2014

**Authors:** Zhou Jiang-jun, Zhao Min, Yan Ya-bo, Lei Wei, Lv Ren-fa, Zhu Zhi-yu, Chen Rong-jian, Yu Wei-tao, Du Cheng-fei

**Affiliations:** 1 Zhou Jiang-jun, MM, Department of Orthopedic, The 184th Hospital of Chinese PLA, Yingtan 335000, Jiangxi Province, China.; 2 Zhao Min, MM, Department of Orthopedic, The 184th Hospital of Chinese PLA, Yingtan 335000, Jiangxi Province, China.; 3 Yan Ya-bo, MD, Department of Orthopedic, Xijing Hospital, the Fourth Military Medical University, Xi’an 710032, Shannxi Province, China.; 4 Lei Wei, MD, Department of Orthopedic, Xijing Hospital, the Fourth Military Medical University, Xi’an 710032, Shannxi Province, China.; 5 Lv Ren-fa, MM, Department of Orthopedic, The 184th Hospital of Chinese PLA, Yingtan 335000, Jiangxi Province, China.; 6 Zhu Zhi-yu, MB, Department of Orthopedic, The 184th Hospital of Chinese PLA, Yingtan 335000, Jiangxi Province, China.; 7 Chen Rong-jian, MB, Department of Orthopedic, The 184th Hospital of Chinese PLA, Yingtan 335000, Jiangxi Province, China.; 8 Yu Wei-tao, MM, Department of Orthopedic, The 184th Hospital of Chinese PLA, Yingtan 335000, Jiangxi Province, China.; 9 Du Cheng-fei, MD, School of Biological Science and Medical Engineering, Beihang University, Beijing 100191, China.

**Keywords:** Biomechanics, Bone healing model, Finite element analysis, Femur fracture, Intramedullary pin

## Abstract

***Objective:*** Finite element analysis was used to compare preoperative and postoperative stress distribution of a bone healing model of femur fracture, to identify whether broken ends of fractured bone would break or not after fixation dislodgement one year after intramedullary nailing.

***Method*** *s: *Using fast, personalized imaging, bone healing models of femur fracture were constructed based on data from multi-slice spiral computed tomography using Mimics, Geomagic Studio, and Abaqus software packages. The intramedullary pin was removed by Boolean operations before fixation was dislodged. Loads were applied on each model to simulate a person standing on one leg. The von Mises stress distribution, maximum stress, and its location was observed.

***Results*** *:* According to 10 kinds of display groups based on material assignment, the nodes of maximum and minimum von Mises stress were the same before and after dislodgement, and all nodes of maximum von Mises stress were outside the fracture line. The maximum von Mises stress node was situated at the bottom quarter of the femur. The von Mises stress distribution was identical before and after surgery.

***Conclusion*** *:* Fast, personalized model establishment can simulate fixation dislodgement before operation, and personalized finite element analysis was performed to successfully predict whether nail dislodgement would disrupt femur fracture or not.

## INTRODUCTION

In the clinic, bone healing after fracture is typically identified by an experienced clinician by reading a common plane film to examine fracture line and callus growth. Whether the internal fixation device should be dislodged or not is assessed simultaneously. Because of the rapid development of computer hardware and progress of software technology, three-dimensional reconstruction and corresponding finite element mechanical analysis can now be done on personal computers using the Windows operating system.^1^^-^^5^

In this study, three-dimensional reconstruction software Mimics and finite element analysis software Abaqus were used to analyze preoperative and postoperative computed tomography (CT) data of femoral fracture patients due to receive device dislodgement during their 1-year follow-up, to compare preoperative and postoperative stress distribution of the model by personalized finite element analysis, and, to determine bone healing conditions and provide evidence for device dislodgement.

## METHODS

***Equipment and software: ***The main equipment used included a 64-row, 128-slice volumetric CT scanner (Lightspeed VCT; GE Healthcare, Waukesha, WI, USA) and Windows 7 Ultimate 64-bit computer (CPU I7-Q720/1.6GHz, memory 4 G, hard disc 320 GB, video card NVIDIA Quadro FX 880 M/1 G) running Mimics 10.01 (Materialise, Belgium), Geomagic Studio 12.0 (Geomagic, USA), and Abaqus 6.10 (Dassault Systèmes, France).

***Construction of three-dimensional finite element models of the femur before and after surgery:***

***Model construction: ***A male volunteer with middle and distal femur fractures who underwent intramedullary nailing (165 cm height and 70 kg weight) was selected in this study at one year after internal fixation. X-ray analysis had confirmed that the internal fixation device could be dislodged. The femur of the affected extremity received CT scanning before and after internal fixation device dislodgement with scanning conditions: 120 kV, 250 mA, 0.625 mm slice thickness, and 3-second slice acquisition time. Data were stored using the Dicom 3.0 standard. Dicom format default images were introduced into Mimics software. Threshold values were set according to the Bone (CT) Scale in Mimics. Gray values of the intramedullary nail were between 1800 and 3071. Through masking, each slice was erased and drawn. Hollows were filled in using contour lines. Three-dimensional models were reconstructed using Optimal, a setting in Mimics. An ASCII stereolithography (STL) file of the femurs was imported into Geomagic Studio. After deleting something resembling a nail in shape, reducing noise, rapidly smoothing, loosening, and locally removing, a surface mesh was output in STL format, converted to an Inp file, input into Abaqus, and converted to a fitted mesh. During preoperative model processing, the smoothed skeleton surface mesh and intramedullary nail surface mesh underwent Boolean operations, followed by meshing.

***Material attribute assignment: ***After meshing, the file was input into Mimics. Elastic modulus assignment was done according to empirical formulae^6^^,^^7^: *Density *= −13.4 + 1017 × [*Grayvalue*], *E-Modulus *= −388.8 + 5925 × [*Density*], and Poisson’s ratio = 0.3,^8^ as materials contain 10 types.^9^ When density was negative, the elastic modulus was set as 1,000 Pa, and Poisson’s ratio as 0.3.

***Finite element analysis and data*** ***acquisition: ***Nonlinear buckling analysis was used on the finite element models before and after intramedullary nail dislodgement. Static load testing was done on all nodes on the surface of a 3-cm diameter region above the femoral head. Load values were three and nine times body weight, ^9^ *i.e.* 700 N and 6300 N in the direction of gravity. All nodes on the surface of a region at 1 cm below the condyles of the femur were constrained, with 0 degrees of freedom. Stress distributions were directly revealed by plotting stress nephograms.

## RESULTS

**Fig.1:**
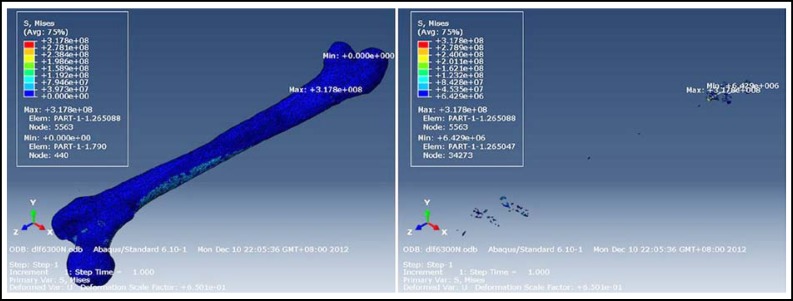
The maximum and minimum von Mises stress of material all and material 08 under nine times weight load before operation

**Fig.2:**
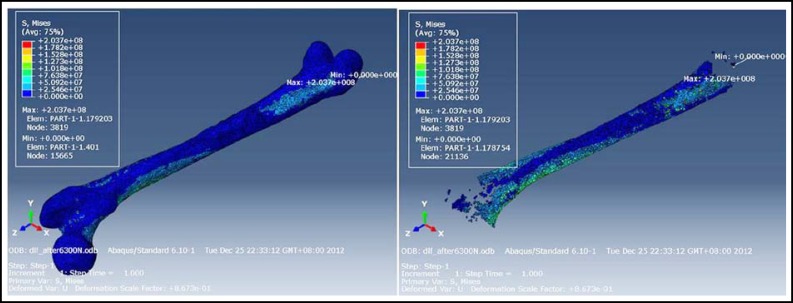
The maximum and minimum von Mises stress of material all and material 08 under nine times weight load after operation

The number of preoperative meshing nodes was 54,345, with 265,868 tetrahedra. The number of postoperative meshing nodes was 40,055, with 195,999 tetrahedra. The maximum and minimum von Mises stress for different materials are shown in Tables-I and Table-II. Under different stress loads, the regions of maximum and minimum von Mises stress of various types of materials were identical. Material properties are shown in Table-III. Stress nephograms revealed that the maximum von Mises stress of each material was not near the broken ends of fractured bone. The stress was not concentrated surrounding the broken ends of fractured bone. The maximum von Mises stress was at the 1/4 juncture of the middle and distal femur, and preoperative and postoperative results were identical (Figs. 1 and 2). Based on these results, preoperative finite element analysis can be used to decide whether the broken ends of the fractured bone would not break again after internal fixation device dislodgement.

## DISCUSSION

Clinically, bone healing, and whether an internal fixation device should be dislodged after fracture, is typically identified by fracture line and callus growth on common plane film. However, a precise, quantitative method for making the assessment was lacking. It is difficult to judge the condition of bone repair if complex or non-ideal bone healing has occurred.

Finite element analysis, first proposed by Professor Clough from the United States in 1960, is a numerical technique for finding approximate solutions to boundary value problems. Applications of this technique in medicine have mainly been in the analysis of structural mechanics and the characteristics of materials. Finite element model construction, meshing production techniques, and arithmetic methods have recently matured. However, finite element used in Orthopedics mainly focused on development and exploitation of various internal fixation materials, but did not conduct personalized research in a single patient model.^10^^,^^11^

**Table-I:** The maximum and minimum von Mises stress for different materials before operation (Pa).

*Type of material*	*2100 N*		*6300 N*	
	*max*	*min*	*max*	*min*
All	1.059E+08	0.000E+00	3.178E+07	0.000E+00
mat1	1.585E+01	1.145E+00	4.754E+00	3.434E+00
mat2	2.198E+01	3.153E-01	6.594E+01	9.459E-01
mat3	5.422E+06	0.000E+00	1.626E+07	0.000E+00
mat4	7.892E+07	0.000E+00	2.368E+08	0.000E+00
mat5	5.713E+07	0.000E+00	1.714E+08	0.000E+00
mat6	8.612E+07	0.000E+00	2.584E+08	0.000E+00
mat7	1.043E+08	8.080E+05	3.129E+08	2.424E+06
mat8	1.059E+08▲	2.143E+06	3.178E+08▲	6.429E+06
mat9	4.808E+07	3.053E+06	1.442E+08	9.160E+06
mat10	4.747E+07	3.081E+06	1.424E+08	9.243E+06

▲: The maximum von Mises stress.

**Table-II:** The maximum and minimum von Mises stress for different materials after operation (Pa).

*Type of material*	*2100 N*		*6300 N*	
	*max*	*min*	*max*	*min*
All	7.122E+07	0.000E+00	2.137E+08	0.000E+00
mat1	4.233E+00	5.879E-01	1.270E+01	1.764E+00
mat2	4.716E+00	1.281E+00	1.415E+01	3.843E+00
mat3	6.195E+00	1.168E+00	1.859E+01	3.504E+00
mat4	1.181E+02	0.000E+00	3.544E+02	0.000E+00
mat5	4.211E+07	0.000E+00	1.263E+08	0.000E+00
mat6	5.114E+07	0.000E+00	1.534E+08	0.000E+00
mat7	6.283E+07	0.000E+00	1.885E+08	0.000E+00
mat8	7.122E+07▲	4.254E-21	2.137E+08▲	1.276E-20
mat9	6.696E+07	6.496E+05	2.009E+08	1.949E+06
mat10	5.894E+07	1.346E+06	1.768E+08	4.038E+06

▲: The maximum von Mises stress.

**Table-III:** The material properties before and after operation

*Type of material*	*Preoperation*			*Postoperation*		
	*Density (mm3)*	*Elastic modulus (Pa)*	*Poisson’s ratio*	*Density* *(mm3)*	*Elastic modulus (Pa)*	*Poisson’s ratio*
mat1	-8.186E+05	1.000E+03	0.3	-8.989E+05	1.000E+03	0.3
mat2	-4.047E+05	1.000E+03	0.3	-6.139E+05	1.000E+03	0.3
mat3	9.191E+03	5.446E+07	0.3	-3.290E+05	1.000E+03	0.3
mat4	4.231E+05	2.507E+09	0.3	-4.398E+04	1.000E+03	0.3
mat5	8.370E+05	4.959E+09	0.3	2.410E+05	1.428E+09	0.3
mat6	1.251E+06	7.411E+09	0.3	5.260E+05	3.116E+09	0.3
mat7	1.665E+06	9.864E+09	0.3	8.110E+05	4.850E+08	0.3
mat8	2.079E+06	1.232E+10	0.3	1.096E+06	6.494E+09	0.3
mat9	2.493E+06	1.470E+02	0.3	1.381E+06	8.182E+09	0.3
mat10	2.906E+06	1.722E+10	0.3	1.666E+06	9.871E+09	0.3

Skeletal bones typically contain compact and cancellous bone. Different materials are compared and the rationality of novel structures was assessed by the elastic modulus and Poisson’s ratio.^12^^-^^14^ The skeleton is a composite of various materials, and its functions cannot be fully revealed by reductive studies. In this study, Mimics, Geomagic Studio, and Abaqus software were used to perform model construction, smoothing, and material assignment. The assignment method was defined using an empirical formula.^6^^,^^7^ Mature modeling techniques, enhanced operating interfaces, and rapid processing speeds can be used to realize personalization in modeling,^15^^,^^16^ allowing the precise data of each patient to be obtained and used. The methods of calculating yield strength of cancellous bone, cortical bone, and callus are different, and the results are different in the different stages of callus formation. The computational procedure would be very complicated if we calculate corresponding yield strength and safety factor according to such conditions, and manual assignment has been commonly used for similar calculations.^17^^,^^18^ Callus measurement and calculation are only in the animal experiment stage.^19^^,^^20^ Weis^21^ used micro-CT and finite element analysis to investigate callus, reporting that finite element analysis was appropriate for longitudinal and therapeutic effects, but only micro-CT could be used during the early stage of bone healing.

How to carry out personalized construction in a bone healing model? We considered that in callus structure, with prolonged time, bone mass is deposited in the callus, bone density increases, and the CT value is also increased. Therefore, we tried to look over stress distribution in accordance with material type (i.e. density classification). That is, sclerotin with similar density was considered having equal yield strength, and assigned an equivalent material property. The callus with high density was assigned with high elastic modulus, which allowed for the personalization of bone healing models.

Table-III shows that the number of materials with negative values after surgery was more than that before surgery. Moreover, there were clearly more preoperative high-density values than after surgery. This is probably due to imaging artifacts from the steel plate before surgery, which increased the CT values of surrounding sclerotin. This likely caused the large difference in pre- and postoperative maximum stress values by finite element analysis. The maximum stress bias was about 48.7%. Although the bias value was high, this study demonstrated that preoperative and postoperative stress concentration points were not at the broken ends of fractured bone, but at the 1/4 juncture of the middle and distal femur. Preoperative and postoperative stress distributions were identical, which was possible because the intramedullary nail went through >80% of the femur, and its artifact affected the CT values of the femur. Thus, we believe that the preoperative and postoperative results are truly identical. The preoperative results could also be used to decide whether the internal fixation device should be dislodged or not. After surgery, standing upright cannot lead to further breakage of the fractured zone.

In this study, personalized model construction of a single patient was done to simulate fixation dislodgement using finite element analysis. Model construction and arithmetic were conducted on a personal, mobile workstation. A skilled engineer constructed the single model and completed finite element analysis in 4–5 hours. This time has decreased, but it remains too long. We will try to improve the operational approach or test method, decrease judgment time, and enhance efficiency for practical application.

## Notes

***Declaration of conflicting interests:*** The author(s) declared no potential conflicts of interest with respect to the research, authorship, and/or publication of this article.

## References

[1] Taylor WR, Roland E, Ploeg H, Hertig D, Klabunde R, Warner MD (2002). Determination of orthotropic bone elastic constants using FEA and modal analysis. J Biomech.

[2] Blemker SS, Asakawa DS, Gold GE, Delp SL (2007). Image-based musculoskeletal modeling: applications, advances, and future opportunities. J Magn Reson Imaging.

[3] Cimerman M, Kristan A (2007). Preoperative planning in pelvic and acetabular surgery: The value of advanced computerised planning modules. Injury.

[4] Zhou JJ, Lei W, Wu ZX, Yan YB, Han BJ (2010). Optimal design of ball-socket on artificial cervical joint complexity by finite element analysis. J Med Biomech.

[5] Ren SH, Liu HT, Liu XY, Zhang ZF, Qi XJ (2011). Establishment of Three-dimensional Finite Element Model of the Menisci. Progress in Modern Biomedicine.

[6] El’Sheikh HF, MacDonald BJ, Hashmi MSJ (2003). Finite element simulation of the hip joint during stumbling: A comparision between static and dynamic loading. J Mat Proc Technol.

[7] Jiang HB, Ge SR (2007). Human femur finite element analysis based on CT scan data. Eng Mech.

[8] Lengsfeld M, Schmitt J, Alter P, Kaminsky J, Leppek R (1998). Comparision of geometry-based and CT voxel-based finite element modelling and experimental validation. Med Eng Phys.

[9] Zhang GD, Liao WJ, Tao SX, Mao WY, Chen JQ, Zheng XH (2009). Methods for material assignment of finite element analysis with femurs. J Clin Rehabilitative Tissue Eng Res.

[10] Burkhart TA, Andrews DM, Dunning CE (2013). Finite element modeling mesh quality, energy balance and validation methods: A review with recommendations associated with the modeling of bone tissue. J Biomech.

[11] Lin D, Li Q, Li W, Swain M (2009). Dental implant induced bone remodeling and associated algorithms. J Mech Behav Biomed Mater.

[12] Wei GJ, Yang H, Lin LJ (2012). Numerical modeling of impact character in proximal femur after hip resurfacing. Chinese J Orthopaedic Trauma.

[13] Yuan GX, Wang L, Zhang WB, Shen YH, Chen B (2012). The finite element analysis of intramedullary and extramedullary fixation in osteoporotic patients with intertrochanter fracture of femur. Chinese J Orthopaedic Trauma.

[14] Du CL, Ma XL, Ma JX (2012). Influence of anteversion angles on stress distributions of the proximal femur after femoral neck fracture fixation: A finite element analysis. J Med Biomech.

[15] Wang JY, Zhang QH, Colin L, Liu Q, Ding XM, Guo ZX (2010). The establishment and significance of three-dimensiional finite element model of pelvis: verified by experiment test (the first part). Chinese J Orthopaedics.

[16] Wang JY, Zhang QH, Colin L, Liu Q, Ding XM, Guo ZX (2010). The establishment and significance of three-dimensiional finite element model of pelvis: verified by experiment test (the second part). Chinese J Orthopaedics.

[17] Zhang XL, Wang XP, Yu XG, Zeng BF (2007). A comparison of biomechanical environments of fracture healing: active weight-bearing vs. passive loading. Chinese J Orthopaedic Trauma.

[18] Shen H, Chen G, Wang JG, Wang YJ (2001). 3-D finite element stress and vibration analysis of rabbit ulna and callus under vibration stress. Chinese J Biomed Eng.

[19] Steiner M, Claes L, Simon U, Ignatius A, Wehner T (2012). A computational method for determining tissue material properites in ovine fracture calluses using electronic speckle pattern interferometry and finite element analysis. Med Eng Phys.

[20] Vetter A, Liu Y, Witt F, Manjubala I, Sander O, Epari DR (2011). The mechanical heterogeneity of the hard callus influences local tissue strains during bone healing: afinite element study based on sheep experiments. J Biomech.

[21] Weis JA, Granero-Moltó F, Myers TJ, Longobardi L, Spagnoli A, Miga MI (2012). Comparison of microCT and an inverse finite element approach for biomechanical analysis: results in amesenchymal stem cell therapeutic system for fracture healing. J Biomech.

